# Trustworthy Clinical Practice Guidelines of Pharmacological Treatments for Hospitalized Patients With COVID-19—A Scarce Resource

**DOI:** 10.1001/jamanetworkopen.2021.38621

**Published:** 2021-12-01

**Authors:** Karina W. Davidson, Catherine M. Alfano, Felicia Hill-Briggs

**Affiliations:** Institute of Health System Science, Feinstein Institutes for Medical Research, Northwell Health, Manhasset, New York, New York.

Burns and colleagues^1^ report the trustworthiness ratings, based on National Academy of Medicine standards, of 32 existing clinical practice guidelines for the use of pharmacological treatments for hospitalized patients with COVID-19. Clinical practice guidelines that are objective, high quality, and credible are always essential.^2^ Methodologically rigorous guidance for clinicians is of paramount importance during a worldwide pandemic, with the rapid emergence of conflicting trial results and the largely unknown benefits and harms of many proposed treatments. To rate the 32 systematically identified clinical management guidelines that exist for clinicians treating hospitalized patients with COVID-19, Burns et al^1^ used the Agency of Healthcare Research Quality NEATS (National Guideline Clearinghouse Extent of Adherence to Trustworthy Standards) instrument.^3^ The results of this quality review of existing COVID-19 clinical management guidelines are sobering.

Discerning the pharmacological treatments that help and those that harm patients with COVID-19 has been a herculean task for clinicians dealing with severely ill patients hospitalized during the pandemic. Clear guidance for clinicians is desperately needed. Yet, only 5 of the 32 guidelines^4–8^ reviewed by Burns et al^1^ engaged in best guideline developments or reporting practices, leaving 27 guidelines (or 84%) that were of low quality according to the NEATS instrument ratings. Many of these low-quality guidelines conflicted in their recommendations; for example, the use of hydroxychloroquine was recommended by 7 low-quality guidelines and recommended against by 3 low-quality guidelines.^1^ Lopinavir plus ritonavir was recommended by 6 low-quality guidelines and recommended against by 2 low-quality guidelines.^1^ When clinicians receive conflicting recommendations about using specific treatments for patients with COVID-19, patient progress is harmed, conflicts among treating multispecialty care teams ensue, and clinical opinions and single-case outcomes continue to influence clinical practice. Most existing clinical management guidelines about treating COVID-19 contribute to rather than solve these problems. One of the many troubling estimates from the article by Burns et al^1^ is how few clinical management guidelines report conflicts of interest or funding sources. Missing these elements does not confirm suspect funding or nefarious influences, but it raises the concern and invites questioning of the objectivity and trustworthiness of the guidelines.

Ideal clinical management guidelines state, at minimum, existing conflicts of interest, funding sources, and methodological approaches, and they report on the quality and strength of the evidence and the benefits and harms of the recommendation. Five clinical management guidelines in the study by Burns et al^1^ met most of the NEATS instrument trustworthy elements.^4–8^ Each reviewed different classes of pharmacological interventions for different purposes, such as preventing venous thromboembolism or mitigating rapid clinical deterioration in mechanically ventilated patients with COVID-19. However, where high-quality guidelines reviewed the same pharmacological treatments, they generally agreed with each other despite a range of publication dates for each guideline. For example, 3 of 3 high-quality guidelines recommended against the use of lopinavir plus ritonavir,^4,6,8^ and 3 of 3 recommended low-dose corticosteroid treatment in severely ill adults with COVID-19.^4,6,8^ Similarly, 2 of 2 recommended prophylaxis against developing later venous thromboembolism,^4,7^ and 2 of 2 recommended vasopressors or inotropes for clinical management.^4,8^ Yet, even these months-old recommendations may not provide the most current guidance on the best management of hospitalized patients with COVID-19. Evidence-based pharmacological treatment of hospitalized patients with COVID-19 is transforming rapidly, with many trials reporting results each month. Two particularly useful clinical practice guidelines—both from the World Health Organization—are living reviews of current evidence.^5,6^

Unlike the clinical management guidelines reviewed, the article by Burns et al^1^ itself—which is based on a clear and thorough systematic review—is highly rigorous. The conclusions, while highlighting the methodological weaknesses of the guidance available to treat hospitalized patients with COVID-19, are clear, and the suggestions offered by this review to improve future guideline development are compelling. Ongoing high-quality efforts to disseminate guidance is needed as the creation of trustworthy clinical management guidelines is one of the highest priorities, especially during medical and public health crises and when evidence bases are quickly accumulating.
